# Incretins in the Therapy of Diabetic Kidney Disease

**DOI:** 10.3390/ijms222212312

**Published:** 2021-11-15

**Authors:** Agnieszka Przezak, Weronika Bielka, Andrzej Pawlik

**Affiliations:** Department of Physiology, Pomeranian Medical University in Szczecin, 70-111 Szczecin, Poland; agn-prze@wp.pl (A.P.); weronika.bielka@wp.pl (W.B.)

**Keywords:** diabetes, diabetic kidney disease, incretins, therapy

## Abstract

Diabetic kidney disease is a microvascular complication that occurs in patients with diabetes. It is strongly associated with increased risk of kidney replacement therapy and all-cause mortality. Incretins are peptide hormones derived from the gastrointestinal tract, that besides causing enhancement of insulin secretion after oral glucose intake, participate in many other metabolic processes. Antidiabetic drug classes, such as dipeptidyl peptidase 4 inhibitors and glucagon-like peptide receptor agonists, which way of action is based on incretins facility, not only show glucose-lowering properties but also have nephroprotective functions. The aim of this article is to present the latest information about incretin-based therapy and its influence on diabetic kidney disease appearance and progression, point its potential mechanisms of kidney protection and focus on future therapeutic possibilities bound with these two antidiabetic drug classes.

## 1. Introduction

Incretins are peptide hormones derived mainly from the gastrointestinal tract which are responsible for the so-called ‘incretin effect’. This is defined as the enhancement of the amount of insulin secreted after oral glucose intake in comparison with the level of insulin secreted after intravenous glucose infusion resulting in the same glycaemia [1]. This phenomenon is known to be crucial to the regulation of postprandial glucose increase, being responsible for up to 70% of insulin secretion in healthy individuals [1]. Therefore, glucose-dependent insulinotropic polypeptide (or, as previously called, gastric inhibitory polypeptide) (GIP) and glucagon-like peptide 1 (GLP1), hormones mostly involved in the incretin effect, are still under deep investigation which could point to possible methods of treating diseases of civilization [2]. The first medical indication of incretin-based therapy was the management of type 2 diabetes (T2D), as it results from the functional failure of β-cells triggered by insulin resistance [3]. It was demonstrated that GLP1 infusion, but not GIP administration, may restore the proper incretin effect in T2D-affected patients in whom it is reduced or even absent [4,5]. This resulted in the development of two antidiabetic drug classes—the glucagon-like peptide receptor (GLP1R) agonists (GLP1RAs) and the dipeptidyl peptidase 4 inhibitors (DPP4is). From then on, scientists’ attention has been drawn to the additional properties of incretin-based therapy, such as treatment of overweight and obesity, nephroprotective features, the reduction of cardiovascular risk, or beneficial effects in liver diseases and neurodegenerative disorders.

Diabetic kidney disease (DKD) is a microvascular complication that develops in approximately 30% of patients with type 1 diabetes (T1D) and approximately 40% of patients with T2D [6,7]. Nowadays, it has become the main reason for end-stage renal disease (ESRD) in the United States [6]. The presence of DKD is strongly associated with the excess risk of all-cause and cardiovascular disease mortality for patients with diabetes [8]. Hyperglycaemia and other metabolic changes coexisting with diabetes cause glomerular hypertrophy, glomerulosclerosis, tubulointerstitial inflammation and fibrosis [9]. The next stages of DKD consist of glomerular hyperfiltration, progressive albuminuria, declining glomerular hyperfiltration rate (GFR) and finally ESRD [9]. However, the majority of patients die from infections and cardiovascular diseases before they require kidney replacement therapy (KRT) [9]. Besides the fact that the diagnosis of DKD is frequently based on clinical symptoms and blood sample analysis, it may be precisely identified only by histological examination of the kidney biopsy [9]. Incretin-based therapy may be a key target to delay the occurrence of DKD and its consequences thanks to incretin’s cardio- and nephroprotective properties and ability to reduce inflammation and fibrosis [10]. In this article, we summarize the physiology of incretin hormones and the drugs based on incretins used in T2D treatment. We focus on their role in DKD to underline their potential nephroprotective properties.

## 2. Metabolism of Glucagon-Like Peptide 1

GLP1 is a peptide produced from proglucagon by proprotein convertase subtilisin-kexin type 1 (PCSK1) or type 3 (PCSK3) in a post-translational process in L cells which are located mainly in the terminal ileum and colon [11]. Moreover, GLP1 production has also been documented in neurons within the nucleus of the solitary tract [12]. The secretion of GLP1 is strongly stimulated by the digested products of consumed food, glucose, amino acids and free fatty acids, as well as by bile acids secreted after food intake, via the mechanism connected with intracellular calcium and/or cAMP levels [13,14]. Not only do the nutrients take part in the exocytosis of incretins, but also a lot of other different stimuli contribute to this complicated process, a lot of them not fully understood.

**Figure 1:**
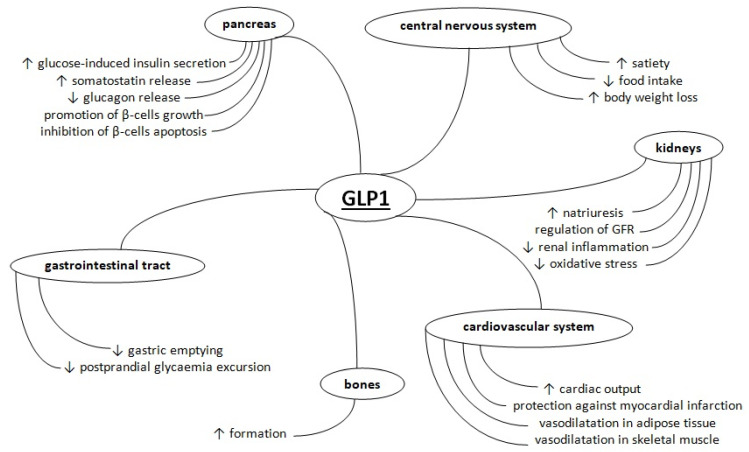
The influence of GLP1 on different tissues.

GLP1 acts through its receptor, GLP1R, one of the class B G protein-coupled receptor families [15]. The receptors are expressed in various tissues, which indicates a huge role of GLP1 in maintaining the homeostasis of the whole organism, related not only to glucose metabolism, but also other aspects of the proper functioning of the human body. They are found in the pancreas, intestine, central and peripheral nervous system, kidneys, heart, lungs, stomach, smooth muscle, adipose tissue and skin [16,17]. Acting through pancreatic GLP1Rs, GLP1 potentiates insulin secretion induced by glucose, as well as promoting growth and inhibiting the apoptosis of β-cells [1,18,19]. It also induces the release of somatostatin from δ-cells and, as a consequence, inhibits the release of glucagon from α-cells [20]. Moreover, GLP1 participates in the mechanism called ‘the ileal brake’ which delays gastric emptying and inhibits intestinal motility via vagal afferent stimulation, which results in a gastroparesis-like situation and a decrease in postprandial glycaemia excursion [21,22]. Incretin also promotes satiety, reduces food intake, and consequently, induces loss of body weight via activation of centres related to food intake in the central nervous system [11,23]. In kidneys, GLP1 reduces the activity of sodium–hydrogen exchanger 3 and thus increases natriuresis [24]. It may have independent nephroprotective and cardioprotective properties—GLP1 increases natriuresis, regulates glomerular filtration rate, declines renal inflammation and oxidative stress, as well as protects against myocardial infarction by activation of pro-survival kinases [25,26]. GLP1 ameliorates cardiac output and promotes vasodilatation in adipose tissue and skeletal muscle, increasing insulin-stimulated glucose uptake in muscle [27,28]. What is more, GLP1 is also able to increase bone formation in overweight or obese people [29]. The actions of GLP1 are presented in Figure 1.

**Figure 2:**
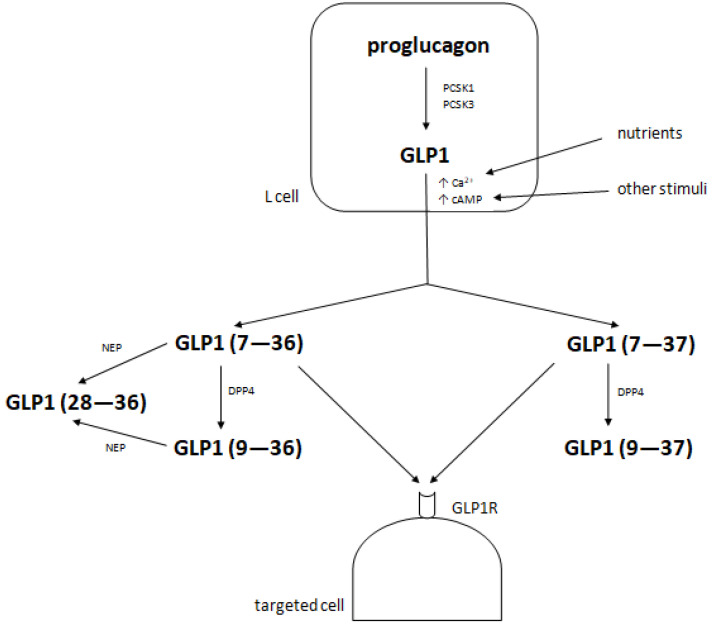
The metabolism of GLP1. A description is given in the text above.

GLP1 circulates in two bioactive forms: carboxy-terminal-amidated GLP1 (7-36) and non-amidated (or glycine-extended) GLP1 (7-37), which are cleaved to produce GLP1 (9-36) and GLP1 (9-37), respectively, within a few minutes by the enzyme dipeptidyl peptidase 4 (DPP4) [16,30]. DPP4 is a pleiotropic enzyme that occurs in two forms—as a membrane-bound protein and as a soluble circulating protein. It inactivates not only incretins, but also a wide range of hormones, chemokines, peptides, substance P and much more [31]. It has been suggested that DPP4 plays a role in the modulation of immune cell functioning, including migration and proliferation [32]. The products of GLP1 degradation do not show any regulatory properties in glucose homeostasis through the GLP1Rs as they are not ligands for these receptors [33,34]. Another enzyme, neutral endopeptidase (or neprilysin, NEP), produces GLP1 (28-36) from GLP1 (7-36) or GLP1 (9-36), which has been shown to increase glucose utilization by the liver and decrease glycaemia [35]. Only a small amount of active GLP1 reaches targeted organs, exerts its actions there and then is cleaved with its metabolites in the kidneys through glomerular filtration and renal extraction [11,36]. The metabolism of GLP1 is presented in Figure 2.

### 2.1. Glucagon-Like Peptide 1 Receptor Agonists—Drugs Showing the Beneficial Effects of Native GLP1

Due to the rapid degradation of GLP1 by DPP4 and thus its short half-life, it was necessary to develop different strategies which could enable the potency of incretins to be used in the treatment of diabetes. One of them is based on exendin-4 which is a naturally occurring substance in the saliva of the lizard *Heloderma suspectum*. Its structure is homologous enough to native GLP1 to activate GLP1R, but simultaneously not so similar as to become degraded by DPP4, so exendin-4 has become a prototype for the structure of exenatide and lixisenatide [37]. However, antidiabetic agents based on exendin-4 still show susceptibility to renal elimination which results in a relatively short half-life, intermittent activation of GLP1Rs and the need for frequent injections. Therefore, it has been proposed to modify human GLP1 in a way that protects it from DPP4 degradation and minimizes renal clearance. Albiglutide and semaglutide are covalently bound and liraglutide is noncovalently bound to albumin, whereas dulaglutide is bound to antibody fragment crystallizable (Fc) domains of immunoglobulin G. Moreover, exenatide has been incorporated in an injectable microsphere which allows it to be slowly released and is known as exenatide extended-release (XR) [30]. These alterations mean that GLP1RAs need to be injected only once a week (except liraglutide which is a once-daily agent). One of the milestones in the development of GLP1RAs was the production of the active substance in an oral form, as it has low bioavailability as a pill [38]. The solution used in oral semaglutide is a fusion with a carrier—sodium N-(8-[2-hydroxybenzoyl] amino) caprylate (SNAC). SNAC increases localized pH levels and causes a pepsin-inhibiting effect in the stomach, which protects semaglutide from degradation and enhances its absorption [39]. Moreover, SNAC also improves the durability of the active substance [38].

GLP1RAs exert similar effects to those of endogenous GLP1. They promote insulin secretion, decrease glucagon release and improve β-cell function, influencing insulin resistance and increasing insulin sensitivity, as well delaying gastric emptying [40,41]. The beneficial impact on glucose homeostasis is seen in the reduction of haemoglobin A_1c_ (HbA_1c_) and fasting glucose level [42,43]. Moreover, this class of drugs may contribute to weight loss, not only through their ability to slow gastric emptying but also through promoting satiety [44,45].

There are some differences between substances resulting from their half-lives. Short-acting GLP1RAs, including exenatide and lixisenatide, activate GLP1Rs in an intermittent way, imitating the action of native GLP1. This results in the preservation of the ability to slow gastric emptying and influence the postprandial glucose rise which is higher than that observed for long-acting GLP1RAs [46,47,48,49]. The long-acting GLP1RAs include liraglutide, exenatide XR, albiglutide, dulaglutide and semaglutide which activate GLP1Rs continuously. They have a superior impact on fasting glucose level and HbA_1c_ than short-acting agents but do not preserve the ability to delay gastric emptying, probably because of tachyphylaxis resulting from consistent receptor activation [50,51]. No difference in body weight reduction is seen between short- and long-acting agonists [16]. A comparison of these two classes is presented in Table 1.

It is worth highlighting that incretin use may improve the response to therapeutic inertia in patients with diabetes. Therapeutic or clinical inertia is defined as a delay in treatment intensification despite suboptimal glycaemic control [52]. This phenomenon causes patients that do not achieve goals of diabetes treatment, spend more time in hyperglycaemia and have a greater risk of diabetes complications. Incretins can help to reduce inertia thanks to their pleiotropic mechanisms and ability to influence not only glucose levels but also body weight or blood pressure, as well as their low risk of hypoglycaemia and no dangerous adverse effects.

The most common side effects of GLP1RA-based treatment are gastrointestinal symptoms, such as nausea, vomiting and diarrhoea, and injection site reactions. The risk of hypoglycaemia during GLP1RA therapy is very low, as the effects of the agents are glucose-dependent [42]. GLP1RAs are thought to be safe and efficient anti-diabetic drugs, and they draw scientists’ attention more and more because of their possible additional properties.

### 2.2. Dipeptidyl Peptidase 4 Inhibitors—Drugs Promoting the Action of Native GLP1

The next strategy to extract beneficial properties from native incretins is based on the inhibition of DPP4, which reduces GIP and GLP1 degradation and increases the levels of endogenous incretins, promoting their actions [53]. The exact role of DPP4 inhibition in glucose homeostasis regulation is not yet fully understood; it cannot be explained only by prolongation of native GLP1 half-life, probably involving prolongation of the half-life of other incretin hormones and neuropeptides which are regulated by DPP4 as well [54]. The DPP4is include linagliptin, alogliptin, saxagliptin, sitagliptin and vildagliptin which have comparable efficacy in reducing glucose levels [55]. They do not have as strong an effect on gastric motility as GLP1RAs, and they are weight-neutral but have a low risk of hypoglycaemia similar to GLP1RAs [56]. The most common side effects of GLP1RA-based treatment are gastrointestinal symptoms, headache, nasopharyngitis and upper respiratory tract infections [57].

### 2.3. Effects of GLP1RAs and DPP4is in Diabetic Kidney Disease

Besides primary cardiovascular disease outcomes, cardiovascular outcome trials (CVOTs) for some GLP1RAs and DPP4is had secondary kidney disease outcomes. The Liraglutide Effect and Action in Diabetes: Evaluation of Cardiovascular Outcome Results (LEADER) trial enrolled 9340 patients with T2D and high cardiovascular risk [58]; 23% of them had moderate-to-severe chronic kidney disease. The secondary outcomes analysed in this trial were the persistent doubling of the serum creatinine level, new-onset persistent macroalbuminuria, kidney failure, or death due to kidney disease. In the liraglutide treatment group, a reduction in the composite prespecified secondary kidney outcome was observed. After 3.8 years, liraglutide therapy reduced the amount of new-onset nephropathy or prevented worsening nephropathy by 22%. The renal benefit was predominantly driven by a 26% reduction in macroalbuminuria. Compared to placebo, liraglutide slowed a decline in eGFR over time. Moreover, liraglutide treatment was associated with a lower rate of renal outcome occurrence, driven primarily by a reduction of the new onset or persistent macroalbuminuria [59]. The Trial to Evaluate Cardiovascular and Other Long-Term Outcomes with Semaglutide in Subjects with Type 2 Diabetes (SUSTAIN-6) enrolled 3297 patients with T2D, of whom 2735 had established cardiovascular disease and/or chronic kidney disease. A secondary outcome was a composite of new-onset or worsening nephropathy defined as persistent doubling of the serum creatinine level, persistent macroalbuminuria, an eGFR < 45 mL/min/1.73 m^2^, or the need for continuous KRT, that is dialysis or kidney transplant [60]. In the injectable semaglutide treatment group, rates of new-onset or worsening nephropathy were lower than in the placebo group. A 46% reduction in new-onset macroalbuminuria was responsible for the favourable renal outcome [60]. The Exenatide Study of Cardiovascular Event Lowering (EXSCEL) trial assessed once-weekly formulations of exenatide in 14,752 participants with T2D and with or without previous cardiovascular disease [61]. The analysis of prespecified secondary kidney outcomes reported no differences between exenatide and placebo groups [62].

Compared with GLP1RAs, the effects of DPP4is bound to kidney protection are modest and mainly result from reducing albuminuria. From the DPP4i group, only treatment with linagliptin does not require a dose adjustment in the case of GFR lowering. The Cardiovascular and Renal Microvascular Outcome Study with Linagliptin (CARMELINA) trial enrolled 6991 participants with T2D and high risk of cardiovascular or chronic kidney disease [63]; 74% of patients had an eGFR < 60 mL/min/1.73 m^2^ and/or urine albumin-to-creatinine ratio (UACR) > 300 mg/g and about 15% of them had an eGFR < 30 mL/min/1.73 m^2^. The only secondary kidney outcome which was significantly improved with linagliptin treatment was albuminuria progression. Other kidney outcomes, such as a sustained decrease in eGFR of ≥40% from baseline, sustained kidney failure and death due to kidney failure showed no difference between linagliptin and placebo groups [63]. The Saxagliptin Assessment of Vascular Outcomes Recorded in Patients with Diabetes Mellitus-Thrombosis in Myocardial Infraction (SAVOR-TIMI 53) trial involved 16,492 patients with T2D and atherosclerotic cardiovascular disease risk factors [64]. The prespecified kidney composite outcomes defined as a change from baseline in UACR, a new-onset or progressed chronic kidney disease, doubling of serum creatinine level, serum creatinine level > 6.0 mg/dL, initiation of dialysis or kidney transplantation did not differ between saxagliptin and placebo groups besides an improvement in albuminuria outcomes. An overall mean reduction in UACR of 34 mg/g was observed, mainly due to the fact that there was an improvement in UACR in participants with macroalbuminuria [64]. For an eGFR < 30 mL/min/1.73 m^2^, the difference in mean UACR change between the two groups was 245 mg/g [65]. A short summary of trials on incretin-based treatments and their influence on renal outcomes is presented in Table 2.

### 2.4. Effects of Incretin-Based Therapy on the Kidneys

The metabolic changes typical for diabetes cause activation of a pro-inflammatory state. Enhancement of oxidative stress and increased production of advanced glycation end-products (AGEs) are crucial mechanisms associated with hyperglycaemia. Inflammation plays a key role in DKD pathogenesis [9]. Moreover, systemic hypertension and glomerular hyperfiltration lead to haemodynamic abnormalities and damage the vasculature of the glomerulus [9].

Patients with diabetes have increased serum levels of advanced oxidation protein products (AOPPs), which are new markers of protein damage induced by oxidative stress. These compounds may have a pro-inflammatory role and induce apoptosis of podocytes in the kidney. Chronic plasma accumulation of AOPPs has been related to proteinuria, glomerulosclerosis and loss of podocytes. Moreover, AOPPs promote the production of reactive oxygen species, induce NADPH oxidase and activate NF-κB. AOPPs are probably associated with the development of DKD as well [66].

**Figure 3:**
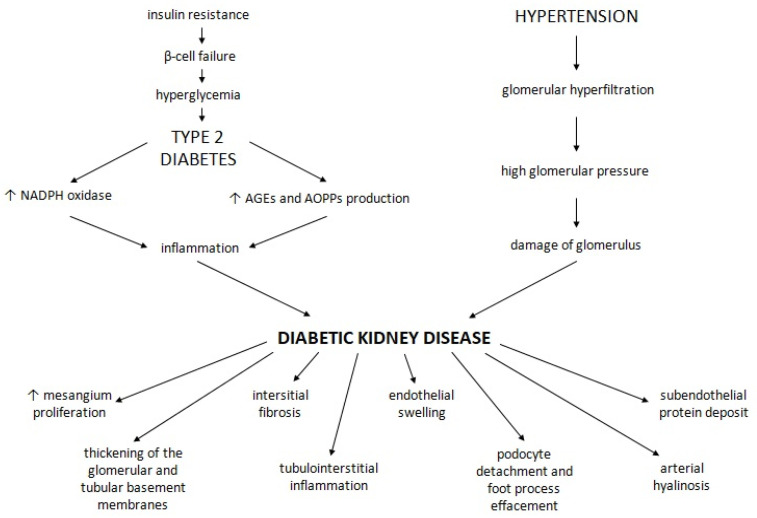
The pathophysiology of diabetic kidney disease. A description is given in the text above.

DKD results in increased matrix expansion, the morphological manifestation of diffuse or nodular proliferation of the mesangium and diffuse thickening of the glomerular and tubular basement membranes [67]. Other features of DKD are interstitial fibrosis, tubulointerstitial inflammation with immune cell infiltration, endothelial swelling with loss of fenestrations, podocyte detachment and foot process effacement, subendothelial protein deposits and arteriolar hyalinosis [68]. The pathophysiology of DKD is presented in Figure 3.

Another important problem is aggressive nephropathy in youth-onset T2D that occurs earlier in life in comparison to nephropathy present in T2D-affected adults or patients suffering from T1D. The young population with T2D presents many additional risk factors of kidney failure, such as obesity, dyslipidaemia, hypertension and inflammation. The main mechanisms involved in the pathogenesis and progression of the DKD in young people are insulin resistance and impaired insulin secretion [69]. Moreover, puberty has a possible influence on the progression of DKD lesions, increasing the production and activity of transforming growth factor-β (TGF-β), a key factor in the development of diabetic renal hypertrophy and nephropathy [70]. Normal changes occurring during puberty, such as an increase in blood pressure, appear in hyperglycaemia due to a physiological decrease of insulin sensitivity, activation of the growth hormone-insulin-like growth factor I axis, or sex steroids production, may intensify diabetic hypertrophy alterations [70]. Furthermore, oestrogen receptors (ERα and ERβ) are involved in insulin secretion and glucose uptake, which may be responsible for gender differences in insulin resistance [71]. Women with metabolic syndrome present reduced muscle ERα expression level, which supports the theory about the protective role of these receptors in the regulation of metabolic homeostasis [72]. Glucose uptake is regulated greatly by insulin-regulated glucose transporter GLUT4 in skeletal muscle. Its expression is diminished by ERβ agonists [73]. ERβ suppresses GLUT4 expression, whereas ERα is a positive regulator of GLUT4 expression [74].

There exists a possibility to detect markers of inflammation and fibrosis in the blood and urine of patients with early diabetes. They may be predictors of DKD and can precede kidney damage by years [68,75,76,77,78]. The occurrence of the inflammatory state promotes an increase in interstitial macrophages and dendritic cells in the kidney. This results in the recruitment of additional monocytes and mast cells from the bone marrow [68,78]. Inflammatory cells release a wide range of pro-inflammatory cytokines and chemoattractant molecules. Moreover, many different signalling pathways are activated, and the expression of adhesion molecules is upregulated [68,75,76,77,78]. The level of uromodulin decreases; on the other hand, the level of fibrinogen α-chain, prothrombin fragments and collagen increases [9].

Incretin drugs influence the main mechanisms involved in DKD development. Predominantly, they lead to the maintenance of normal glucose levels and by reducing hyperglycaemia, decrease the formation of pro-inflammatory AGEs [10]. Intensive glycaemic control also results in a reduction in the hyperglycaemia-induced activity of NADPH oxidase, which affects oxidative stress [79]. Furthermore, better glycaemic control helps to diminish glomerular hyperfiltration and high glomerular pressure [10]. Activation of cAMP by GLP1RAs may lead to a reduction in the expression of the receptor for AGEs, causing antioxidative effects [80].

Treatment with GLP1RAs leads to enhancement of natriuresis and diuresis, associated with increased blood flow and reduced vascular resistance in the kidney as a result of an increase in local nitric oxide production [81,82]. Under physiological conditions, GLP1RAs may induce glomerular hyperfiltration by reducing afferent arteriolar resistance, but in patients with T2D, they can improve renal haemodynamic function [24]. Natriuresis is probably induced by inhibition of sodium–hydrogen exchanger 3 (NHE3). Pharmacological doses of GLP1 or GLP1RAs increase intrarenal cAMP generation and activate protein kinase A by binding to its receptor. This causes subsequent phosphorylation of NHE3 and leads to the inhibition of sodium reabsorption in the proximal tube [83]. Low NHE3 activity increases distal delivery of sodium chloride and affects tubuloglomerular feedback, which can decrease glomerular hyperfiltration and pressure [84]. DPP4is might also inhibit NHE3 activity through a tyrosine kinase signalling pathway or redistribute NHE3 and stimulate NHE3-independent sodium excretion [85,86,87]. This natriuretic response may be mediated by elevated levels of intact stromal cell-derived factor 1α (SDF1α) via the sodium-chloride cotransporter or the epithelial sodium channel in the distal convoluted tubule [88].

Incretin drugs block inflammation and fibrosis—mechanisms that cause structural damage in kidneys. In studies, exendin-4 and liraglutide reduced the production of pro-inflammatory cytokines and decreased the expression of TGFβ1, NF-κB and ICAM1, and reduced oxidative stress and kidney infiltration by macrophages [89,90]. Incretin treatment attenuates the pro-inflammatory response by reducing inflammatory cell invasion, blocking the activation of the mononuclear phagocyte system and decreasing the production of chemokines, cytokines, adhesion molecules and pro-fibrotic signalling [91,92,93]. In another study, the use of sitagliptin reduced C-reactive protein (CRP) levels in patients with T2D [94]. After 12 weeks of treatment with exenatide, mononuclear cells collected from patients with diabetes presented such anti-inflammatory effects as reduced NF-κB activation, reactive oxygen species generation and mRNA expression of TLR2, TLR4, TNF, IL-1B, MAPK8 and SOCS3 [95]. The treatment also suppressed levels of IL-6, serum amyloid A, matrix metalloproteinase 9 and CCl2 [95]. Treatment with liraglutide led to decreased albuminuria and urine levels of neutrophil gelatinase-associated lipocalin (NGAL) [96]. Induced by exendin-4, the stimulation of cAMP and protein kinase A in human mesangial cells causes proliferation and fibrosis reduction [97]. Some DPP4is also have the potential to upregulate CD4+ regulatory T cells and diminish levels of IL-6, TNF and CRP [94,98,99]. In patients with T2D, treatment with sitagliptin significantly reduces levels of secreted phospholipase-A_2_, soluble ICAM1, E-selectin, CRP, IL-6 and IL-18 [94]. Expression of SDF1α in glomerular podocytes and distal nephrons induced by linagliptin results in reduced progression of glomerulosclerosis, albuminuria, periglomerular fibrosis, podocyte loss and renal oxidative stress [88].

Incretin therapy may also affect the main renal risk factors. Treatment with GLP1RAs results in a reduction in waist circumference and body weight, especially in total body fat, particularly trunk or visceral fat rather than in lean tissue mass [100,101]. The beneficial effect of GLP1RAs is also bound with a modest decrease in systolic blood pressure [82,83]. In a meta-analysis of 60 randomized controlled trials, the reduction in systolic blood pressure was significant with liraglutide and albiglutide and non-significant with exenatide and dulaglutide compared with placebo [102]. Moreover, in patients with T2D, the use of incretins improves fasting and particularly postprandial lipid profiles. A meta-analysis indicated that therapy with GLP1RAs causes mild reductions in the levels of total cholesterol, LDL cholesterol and triglycerides, without improvement in HDL cholesterol levels [103], whereas another meta-analysis of randomized controlled trials for DPP4is showed a mild reduction in total cholesterol level [104].

## 3. Conclusions

Incretin drugs, another class of antidiabetic drugs, which includes GLP1RAs and DPP4is, have a wide range of pleiotropic modes of action. Besides the ability to maintain normoglycaemia, they have the potential to reduce appetite, body weight and hypertension. Moreover, they show cardio- and nephroprotective effects in a different way, not completely associated with reducing hyperglycaemia. Their multidirectional properties and ability to alter the development of diabetic complications, such as diabetic kidney disease, highlight their strong position among other antidiabetic drugs.

DKD is one of the main causes of ESRD, which makes it a serious problem. Among the mechanisms that take part in DKD development are inflammation, fibrosis, oxidative stress, as well as AGEs and AOPPs generation. Incretin drugs used in diabetes treatment influence these mechanisms by reducing pro-inflammatory and pro-fibrotic states. Their action also results in enhanced natriuresis and diuresis, and the depletion of renal risk factors. Further investigation and trials are needed to know the exact mechanism of the nephroprotective potential of incretins. However, it is already clear that incretin-based therapy is beneficial not only in diabetes treatment but also in therapy of its complications, especially DKD.

## Figures and Tables

**Table 1:** Comparison of short-acting and long-acting GLP1RAs.

Type of GLP1RA	Short-Acting GLP1RAs	Long-Acting GLP1RAs
Agents	exenatide lixisenatide	liraglutide exenatide XR albiglutide dulaglutide semaglutide
Activation of GLP1R	intermittent	continuous
Gastric emptying	delaying	no influence due to tachyphylaxis
Postprandial glucose excursion	superior impact	inferior impact
Fasting glucose levels and HbA_1c_	inferior influence	superior influence
Bodyweight reduction	comparable effect	comparable effect

**Table 2:** A short summary of trials on incretin-based treatments and their influence on renal outcomes.

Trial	Agent	Enrolled Patients	Renal Outcomes	Results	References
LEADER	liraglutide	9340 patients with T2D and high cardiovascular risk	- persistent doubling of the serum creatinine level - new-onset persistent macroalbuminuria - ESRD - death due to renal disease	- a 22% reduction in new-onset or worsening nephropathy - a 26% reduction in macroalbuminuria - slowing eGFR decline over time - lower rate of DKD events	[58,59]
SUSTAIN-6	semaglutide	3297 patients with T2D, of whom 2735 had established cardiovascular disease, chronic kidney disease or both	new-onset or worsening nephropathy is defined as: - persistent doubling of the serum creatinine level - persistent macroalbuminuria - an eGFR < 45 mL/min/1.73 m^2^ - the need for continuous KRT (dialysis or kidney transplant)	- a reduction in rates of new-onset or worsening nephropathy - a 46% reduction in new-onset macroalbuminuria	[60]
EXSCEL	exenatide XR	14,752 patients with T2D, of whom 10,782 had previous cardiovascular disease	- change in eGFR - new macroalbuminuria occurrence - effects on renal composite 1 (40% eGFR decline, renal replacement or renal death) - effects on renal composite 2 (renal composite 1 variables plus macroalbuminuria)	- no significant influence on any kidney outcome	[61,62]
CARMELINA	linagliptin	6991 patients with T2D and high cardiovascular or chronic kidney disease risk	- time to first occurrence of adjudicated death due to renal failure, ESRD or sustained 40% or higher decrease in eGFR from baseline	- a significant improvement of albuminuria progression - no significant differences in other kidney outcomes	[63]
SAVOR-TIMI 53	saxagliptin	16,492 patients with T2D who had a history of, or were at risk for, cardiovascular events	- a change from baseline in UACR - a new-onset or progressed chronic kidney disease - doubling of serum creatinine level - serum creatinine level > 6.0 mg/dL - initiation of dialysis - kidney transplantation	- an improvement in albuminuria outcome - no significant differences in other kidney outcomes	[64,65]

## Data Availability

Not applicable.

## Abbreviations

GIP	glucose-dependent insulinotropic polypeptide (previously known as gastric inhibitory peptide)
GLP1	glucagon-like peptide 1
T2D	type 2 diabetes
GLP1R	glucagon-like peptide 1 receptor
GLP1RA	glucagon-like peptide 1 receptor agonist
DPP4	dipeptidyl peptidase 4
DPP4i	dipeptidyl peptidase 4 inhibitor
DKD	diabetic kidney disease
T1D	type 1 diabetes
ESRD	end stage renal disease
GRF	glomerular filtration rate
KRT	kidney replacement therapy
PCSK1	proprotein convertase subtilisin-kexin type 1
PCSK3	proprotein convertase subtilisin-kexin type 3
cAMP	cyclic adenosine monophosphate
NEP	neutral endopeptidase (neprilysin)
Fc	fragment crystallizable
XR	extended release
SNAC	sodium N-(8-[2-hydroxybenzoyl]amino) caprylate
HbA_1c_	haemoglobin A_1c_
CVOT	cardiovascular outcome trial
LEADER trial	the Liraglutide Effect and Action in Diabetes: Evaluation of Cardiovascular Outcome Results trial
eGFR	estimated glomerular filtration rate
SUSTAIN-6 trial	the Trial to Evaluate Cardiovascular and Other Long-Term Outcomes with Semaglutide in Subjects with Type 2 Diabetes trial
EXSCEL trial	the Exenatide Study of Cardiovascular Event Lowering trial
CARMELINA trial	the Cardiovascular and Renal Microvascular Outcome Study with Linagliptin trial
UACR	urine albumin-to-creatinine ratio
SAVOR-TIMI 53 trial	the Saxagliptin Assessment of Vascular Outcomes Recorded in Patients with Diabetes Mellitus—Thrombosis in Myocardial Infraction trial
AGE	advanced glycation end-product
AOPP	advanced oxidation protein product
NADPH	reduced form of nicotinamide adenine dinucleotide phosphate
NF-ĸB	nuclear factor ĸB
TGF-β	transformin growth factor-β
ERα	oestrogen receptor α
ERβ	oestrogen receptor β
GLUT4	glucose transporter type 4
NHE3	sodium-hydrogen exchanger 3
SDF1α	stromal cell-derived factor 1α
ICAM1	intercellular adhesion molecule 1
TLR2	toll-like receptor 2
TLR4	toll-like receptor 4
TNF	tumour necrosis factor
IL-1B	interleukin 1B
MAPK8	mitogen-activated protein kinase 8
SOCS3	suppressor of cytokine signalling 3
CCl2	C-C motif chemokine ligand 2
NGAL	neutrophil gelatinase-associated lipocalin
CRP	C reactive protein
IL-6	interleukin 6
IL-18	interleukin 18
LDL	low density lipoprotein
HDL	high density lipoprotein
